# Trouble at the races

**DOI:** 10.1186/2041-2223-5-14

**Published:** 2014-10-23

**Authors:** Mark A Jobling

**Affiliations:** 1 Department of Genetics, University of Leicester, University Road, Leicester LE1 7RH, UK

## 

Recently, I signed a letter to the New York Times. This is not something I do very often, but I felt strongly about the subject. And it wasn’t just me - so did the other 140 or so signatories, all of them population geneticists or human evolutionary biologists. What aroused the ire of this dusty community of academics? A pop science book by Nicholas Wade, *A Troublesome Inheritance*: *Genes, Race and Human History*[1].

The objection to the book included its use of the population genetics literature to buttress claims for the existence of five major human races. The influential paper [2] that examined the population structure of microsatellite variation in the 1000 or so Human Genome Diversity Panel DNA samples looms large in this argument. When the program STRUCTURE [3] was asked to find five clusters in the data, these corresponded to Africa, Europe plus the Middle East, South/Central Asia, East Asia, the Americas and Oceania. The genetic differentiation of non-admixed human populations results from a serial founder effect from the out of Africa migrations, followed by the influence of isolation by distance. So it’s not surprising that continental groups are differentiable from each other. It doesn’t make them races, but according to *A Troublesome Inheritance* it does. A later study based on genome-wide SNPs [4] is also cited. From this ‘it might be reasonable to elevate the Indian and Middle Eastern groups to the level of major races, making seven in all. But then many more subpopulations could be declared races, so to keep things simple, the five-race, continent-based scheme seems the most practical’. Exactly.

The book also makes much of the role of natural selection in humans, which is repeatedly described as ‘recent, copious and regional’. Examples are given of the *EPAS1* gene and adaptation to altitude in Tibetans [5], lactase persistence, as well as more ancient adaptations involving pigmentation. If these, then why not behaviour? And why not complex behaviour, too, such as industriousness, tribalism or trust? Of course, it’s a shame we don’t have a clue about their genetic bases, but no doubt this will come in time, and if there are genes involved, ‘recent, copious and regional’ selection has no doubt been at work on them. This leads to the notion that there has been genetic adaptation within races involving behavioural genes that govern such traits: Africans are tribal because their genes are adapted to tribalism, and this explains why foreign aid is often ineffective. Let’s face it, there’s a simplistic view here about the genetic architecture of complex traits. The lessons we’ve learned from genome-wide association studies and their difficulties in explaining heritability are not explored. A lot of other population genetics literature is cited along the way, but with cherry-picking – the caveats and alternative explanations are airbrushed out, so Wade can tell the story he wants to tell. This unwillingness to capture disagreement amongst geneticists contrasts with a description of the failure of economic historians to concur about the causes of the Industrial Revolution. Because they can’t agree, we need a new explanation – hey presto, genetic adaptation!

It’s striking that, after its paean to the power of twenty-first century genomics, the book ends up essentially siding with Linnaeus and his 18th century sub-species classifications of *Homo sapiens europeus*, *asiaticus* and *afer*:

• *Europeans*: white, sanguine, muscular, inventive, governed by laws;

• *Asians*: yellow, melancholy, severe, haughty, stingy, governed by opinions;

• *Africans*: black, cunning, phlegmatic, ruled by impulse.

Wade castigates those who claim that race is just a social construct, stating that these deluded folks are leftists and Marxists, and that their views are politically motivated, and not scientific. And he is partly right. Some of the influential writers on this question have indeed been leftists and Marxists. It is difficult, if not impossible, for anyone to entirely divorce their background and political views from their professional life. If they are lucky enough to work on transcriptional control in yeast, for example, this is probably not much of an issue, but human genetics is a different game. By contrast, the heterodox crew who work on the biological reality of race are characterised as ‘courageous’ seekers after truth. But I’d hazard a guess they all have their own social and political baggage, too.

At the beginning of *A Troublesome Inheritance*, we are told that racism and eugenics are Bad Things, that Hitler was a Bad Man, and that ‘opposition to racism is well entrenched’. Many people would beg to differ with that last statement. Racism is alive and kicking (sometimes literally), and this book provides succour for racists by using speculation to support the idea that differences between population groups are in their genes. It also avers that linking the ‘success of the West’ to ‘Caucasian’ genetics is not racist, because ‘there is no assertion of superiority’. But from reading this book that is certainly not the message that comes across. Social psychologists have noted a significant increase in a belief in essential differences between whites and blacks as a result of genetic ancestry testing [6]. *A Troublesome Inheritance*, whatever its author’s motives, will do the same in spades. Its enthusiastic proponents already include some high profile white supremacists and a former Grand Wizard of the Ku Klux Klan.
